# Lipid Exchange between *Borrelia burgdorferi* and Host Cells

**DOI:** 10.1371/journal.ppat.1003109

**Published:** 2013-01-10

**Authors:** Jameson T. Crowley, Alvaro M. Toledo, Timothy J. LaRocca, James L. Coleman, Erwin London, Jorge L. Benach

**Affiliations:** 1 Department of Molecular Genetics and Microbiology, Center for Infectious Diseases, Stony Brook University, Stony Brook, New York, United States of America; 2 Department of Pediatrics, Columbia University, New York, New York, United States of America; 3 State of New York Department of Health, Stony Brook University, Stony Brook, New York, United States of America; 4 Department of Biochemistry and Cell Biology, Stony Brook University, Stony Brook, New York, United States of America; Medical College of Wisconsin, United States of America

## Abstract

*Borrelia burgdorferi*, the agent of Lyme disease, has cholesterol and cholesterol-glycolipids that are essential for bacterial fitness, are antigenic, and could be important in mediating interactions with cells of the eukaryotic host. We show that the spirochetes can acquire cholesterol from plasma membranes of epithelial cells. In addition, through fluorescent and confocal microscopy combined with biochemical approaches, we demonstrated that *B. burgdorferi* labeled with the fluorescent cholesterol analog BODIPY-cholesterol or ^3^H-labeled cholesterol transfer both cholesterol and cholesterol-glycolipids to HeLa cells. The transfer occurs through two different mechanisms, by direct contact between the bacteria and eukaryotic cell and/or through release of outer membrane vesicles. Thus, two-way lipid exchange between spirochetes and host cells can occur. This lipid exchange could be an important process that contributes to the pathogenesis of Lyme disease.

## Author Summary

Lyme disease, the most prevalent arthropod-borne disease in North America, is caused by the spirochete *Borrelia burgdorferi*. Cholesterol is a significant component of the *B. burgdorferi* membrane lipids, and is processed to make cholesterol-glycolipids. Our interest in the presence of cholesterol in *B. burgdorferi* recently led to the identification and characterization of eukaryotic-like lipid rafts in the spirochete. The presence of free cholesterol and cholesterol-glycolipids in *B. burgdorferi* creates an opportunity for lipid-lipid interactions with constituents of the lipid rafts in eukaryotic cells. We present evidence that there is a two-way exchange of lipids between *B. burgdorferi* and epithelial cells. Spirochetes are unable to synthesize cholesterol, but can acquire it from the plasma membrane of epithelial cells. In addition, free cholesterol and cholesterol-glycolipids from *B. burgdorferi* are transferred to epithelial cells through direct contact and through outer membrane vesicles. The exchange of cholesterol between spirochete and host could be an important aspect of the pathogenesis of Lyme disease.

## Introduction

*Borrelia burgdorferi*, the causative agent of Lyme disease [1], [2], is unusual among prokaryotes in that in addition to phosphatidylcholine, phosphatidylglycerol [3]–[7] and many different lipoproteins [4], [5], [8]–[10], it has free cholesterol and cholesterol-glycolipids in its outer membrane (OM). The glycolipids of *B. burgdorferi* are mono-α-galactosyl-diacylglycerol (MGalD), which does not contain cholesterol; cholesteryl-β-D-galacto-pyranoside (CGal); and cholesteryl 6-O-acyl-β-D-galactopyranoside, or cholesteryl 6-O-palmitoyl-β-D-galactopyranoside (ACGal/Bb-GL-1), which contain cholesterol [3], [11]–[14]. The cholesterol-glycolipids constitute a significant portion, 45% [11], of the total lipid content [3], [5], [12], [13], [15]–[18]. *B. burgdorferi* does not have the biosynthetic ability to synthesize cholesterol or any long-chain-saturated and unsaturated fatty acids that are required for growth [6]. As a result, the lipid composition of *B. burgdorferi* reflects that of the culture medium or host animal fluids or tissues [6]. Furthermore, it has been hypothesized that in addition to the activity of galactosyltransferase bb0454, other uncharacterized spirochetal transferases could be responsible for constructing the cholesterol-glycolipids [18]. Important to the pathogenesis of *B. burgdorferi*, ACGal, and to a lesser extent MGalD and CGal, are antigenic [13], [15]–[17], [19]. These glycolipids induce antibody responses throughout all stages of Lyme disease, being most prominent in the late stages [9], [11], [12], [20], [21]. Additionally, we demonstrated that antibodies to the cholesterol-glycolipids cross-react with host gangliosides and antibodies to the gangliosides cross-react with the glycolipids [22], [23]. *Borrelia* lipid antigens can also be presented in the context of CD1d on NKT cells [24]–[29].

Using ultrastructural, biochemical, and biophysical analysis, we previously determined that the cholesterol-glycolipids in the OM of *B. burgdorferi* are constituents of eukaryotic-like lipid raft domains [30]. In eukaryotic cell membranes, lipid rafts are microdomains that are rich in sterols, sphingolipids, and phospholipids with saturated acyl tails that allow for tight packing of these lipids into ordered domains [31], [32]. These cholesterol-rich domains segregate from the disordered membrane domains that contain mostly unsaturated lipids [31], [33]. In addition to the enrichment of specific lipids, lipid-anchored proteins such as glycosyl phosphatidylinositol (GPI) proteins and proteins covalently linked to saturated acyl chains are targeted to lipid rafts [34]. Lipid rafts are important for the segregation of plasma membrane proteins [31]–[33], [35]–[38], and contribute to endocytosis, exocytosis, vesicle formation, and budding [39]–[43]. Furthermore, lipid rafts have been identified as important platforms in cell signaling [33].

The presence of free cholesterol and cholesterol-glycolipids with saturated acyl chains in *B. burgdorferi* creates an opportunity for lipid-lipid interactions with constituents of the lipid rafts in eukaryotic cells. This is of particular interest since *B. burgdorferi* adheres to many different cell types [44], [45]. Lipid-lipid interactions could also facilitate the ability of the spirochete to adhere to many different types of cells [46]–[49] and to cellular and matrix proteins [50]–[52]. Furthermore, exchange of lipids between spirochetes and host cells could be important for cholesterol acquisition by the spirochetes, acting as an important nutritional source. Additionally, acquisition of spirochetal antigens by the cells could result in presentation of these antigens in a manner that would be recognized by the immune response leading to a potential mechanism for cellular damage.

The requirement for cholesterol is important for other bacteria. The presence of a cholesterol glucoside in spirochetes was first identified in *B. hermsii* [53], an agent of relapsing fever. In addition, cholesterol has been documented in the membranes of *Helicobacter*, *Mycoplasma*, *Ehrlichia*, *Anaplasma*, and *Brachyspira* [54]–[60]. It is unknown whether raft-like structures similar to that in *B. burgdorferi* form in these other bacteria. However, acquisition of cholesterol from the plasma membrane of host cells has been documented with *H. pylori*, another prokaryote that has cholesterol in its OM [61], and this organism associates with cholesterol rich areas of the eukaryotic cells [61], [62].

We show here that there is a two-way exchange of lipids between *B. burgdorferi* and eukaryotic cells and that this exchange is accomplished through direct contact with the spirochete as well as contact with outer membrane vesicles (OMV).

## Results

### *B. burgdorferi* attach to HeLa cells and acquire cholesterol

**Figure 1:**
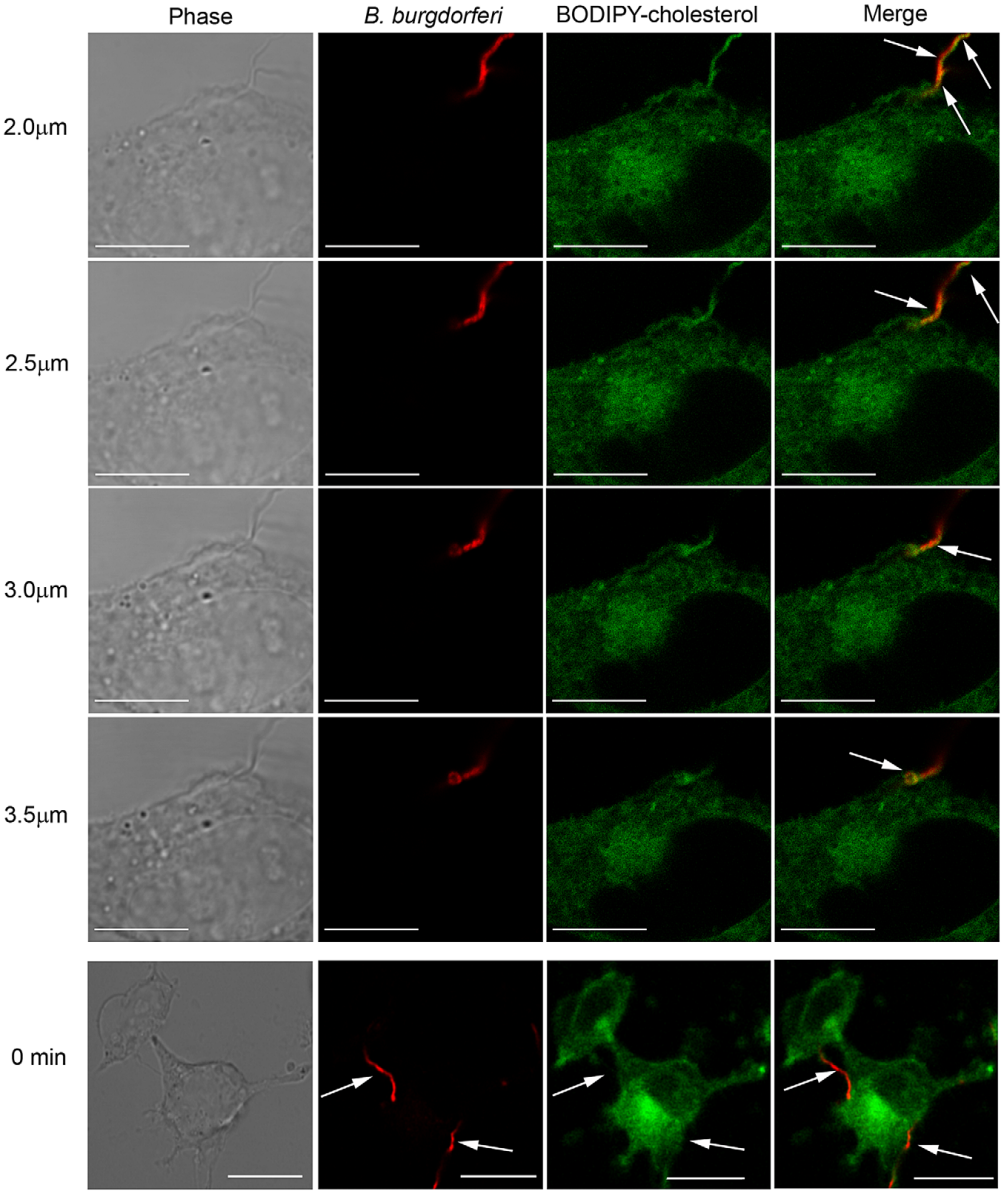
*B. burgdorferi* attach to HeLa cells and acquire cholesterol from the epithelial cell membranes. HeLa cells incubated with BODIPY-cholesterol (green) and washed with MβCD were incubated with *B. burgdorferi* (red) for 1 hr at an MOI of 40∶1. Cells were fixed, stained with CB2 (red) and examined by confocal fluorescence microscopy. Four individual optical sections are shown at 2.0 µm, 2.5 µm, 3.0 µm and 3.5 µm. Confocal micrographs show colocalization (yellow, arrows) of BODIPY-cholesterol (green) and OspB (red) on the spirochete at the point of attachment. Scale bars = 10 µm. Confocal micrographs taken at the start of the experiment, 0 min panels (bottom), show that acquisition of BODIPY-cholesterol (green) has not occurred on the spirochetes (red) because there is no colocalization (yellow). The lack of colocalization is highlighted in the individual panels (arrows). Scale bars = 20 µm.

We first investigated whether *B. burgdorferi* acquires cholesterol through direct contact with HeLa cells using BODIPY-cholesterol. BODIPY-cholesterol is an environment sensitive, lipophilic probe that only fluoresces in hydrophobic, but not aqueous environments [63]–[65]. When *B. burgdorferi* were incubated with HeLa cells labeled with BODIPY-cholesterol at a multiplicity of infection (MOI) of 40∶1, we observed colocalization (yellow) between the BODIPY-cholesterol and *B. burgdorferi* outer membrane protein OspB on the spirochete and at the point of attachment with the HeLa cell (Figure 1, S1 for additional images). Colocalization of BODIPY-cholesterol and the spirochetes was demonstrated in single 0.5 µm Z-slices and showed the uptake of cholesterol by adherent *B. burgdorferi* (Figure 1, S1). Furthermore, BODIPY-cholesterol labeling extended outward from the point of attachment along the length of the spirochete (Figure 1, S1). Acquisition of BODIPY-cholesterol is not detected at the start of the experiment (Figure 1, 0 min panels). HeLa cells do not release BODIPY-cholesterol into the supernatant over the course of the 1 hr coincubation (data not shown); therefore, *B. burgdorferi* most likely acquired BODIPY-cholesterol directly from the labeled HeLa cells and not the supernatant.

### *B. burgdorferi* incorporate the fluorescent cholesterol analog, BODIPY-cholesterol into its outer membrane

**Figure 2:**
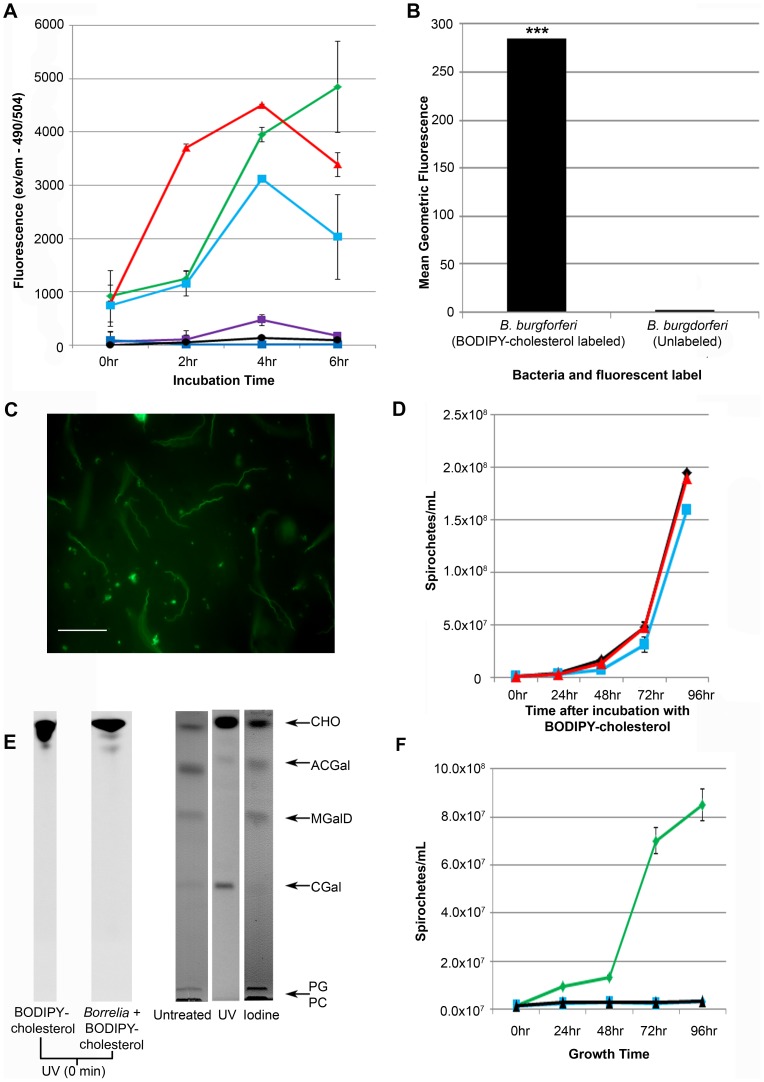
*B. burgdorferi* incorporate BODIPY-cholesterol into its outer membrane as a component of the cholesterol-glycolipids. **A.** Spirochetes were grown for 6 hrs in BSK-II media lacking cholesterol and their fluorescence was measured by spectrophotometry. 4.0 mg/L BODIPY-cholesterol in BSK-II without free cholesterol (green); 0.25% DMSO in BSK-II without free cholesterol (purple); 2.0 mg/L BODIPY-cholesterol BSK-II without free cholesterol (red); 0.175% DMSO in BSK-II without free cholesterol (dark blue); 0.2 mg/L BODIPY-cholesterol in BSK-II without free cholesterol (light blue); 0.0125% DMSO in BSK-II without free cholesterol (black). **B.** *B. burgdorferi* incubated with and without BODIPY-cholesterol were analyzed by flow cytometry. ANOVA ***p<0.001. **C.** Unfixed, live spirochetes grown in the presence of 4.0 mg/L BODIPY-cholesterol for 4 hrs were observed by fluorescent microscopy. The spirochetes did not exhibit morphological or motility defects. Scale bar = 20 µm. **D.** Following incubation of *B. burgdorferi* with 4.0 mg/L BODIPY-cholesterol for 4 hrs, the spirochetes were grown in BSK-II for up to 96 hrs, and there were no significant differences in growth compared to controls. Untreated control (black diamond); labeled with BODIPY-cholesterol (blue square); Incubated with 0.25% DMSO (red triangle). **E.** *B. burgdorferi* were treated and incubated with 4.0 mg/L. *B. burgdorferi* pellets were extracted by Bligh and Dyer lipid extraction and analyzed using a chloroform-methanol (85/15) HPTLC. The HPTLC plate was developed using iodine and exposure to UV light. **F.** *B. burgdorferi* were grown in BSK-II media lacking free cholesterol with BODIPY-cholesterol or cholesterol as the primary source of sterol in the media to assess long term growth and cytotoxicity in the presence of exogenously added sterol. BSK-II (green); 4.0 mg/L BODIPY-cholesterol in cholesterol free BSK-II without serum (blue); 4.0 mg/L cholesterol in cholesterol free BSK-II without serum (black). Experiments **A, B, D,** and **F** represent the mean ± standard error of the mean from three separate experiments.

An important question is whether BODIPY-cholesterol behaves like unlabeled cholesterol in *B. burgdorferi*. To investigate this, we first determined the incorporation of BODIPY-cholesterol onto the membrane of *B. burgdorferi* and its toxicity for the spirochete. To grow *B. burgdorferi* under laboratory conditions, it is necessary to supplement the BSK-II medium with 6% rabbit serum which provides a source of cholesterol (approximately 0.78 mg/L final concentration) bound to apolipoproteins to form lipoprotein complexes [66]. The free cholesterol (0.2 mg/L), and the most accessible to *B. burgdorferi* in BSK-II medium, comes from the CMRL-1066 supplement. Removal of cholesterol from the CMRL-1066 eliminated the free cholesterol found in BSK-II. Two of the three concentrations of BODIPY-cholesterol used in the experiments (2.0 mg/L and 4.0 mg/L) are greater than the 0.78 mg/L of cholesterol derived from rabbit serum in BSK-II and are not bound to apolipoproteins. This suggests that the primary and most readily available source of cholesterol to the spirochetes is the fluorescent cholesterol analog. While *B. burgdorferi* can grow in serum-free formulations [67]–[69], the use of serum remains the most used supplement. Acquisition of the fluorescent probe by the spirochetes was demonstrated by spectrophotometry, by flow cytometry, and by fluorescent microscopy. The spirochetes incorporated BODIPY-cholesterol in a dose and time dependent manner (Figure 2A). At all concentrations measured, the BODIPY-cholesterol was quickly introduced to the membranes of the spirochetes. At 6 hrs of incubation with the fluorescent cholesterol analog, the variability in the levels of incorporation increased. It is possible that at this time, there may be some self-quenching of the BODIPY fluorophore. For this reason, we determined that the most reproducible fluorescence incorporation level was derived from growing the spirochetes for 4 hrs in the presence of 4.0 mg/L BODIPY-cholesterol. It is important to point out that all our incorporation experiments were done at, maximally, 4 hr time periods. Using these optimized conditions, we measured the mean geometric fluorescence of *B. burgdorferi* labeled with BODIPY-cholesterol compared to unlabeled *B. burgdorferi* by flow cytometry. *B. burgdorferi* grown in the presence of BODIPY-cholesterol were significantly more fluorescent when compared to unlabeled bacteria (Figure 2B). Spirochetes grown in DMSO (which is the diluent for BODIPY-cholesterol) do not autofluoresce.

At the concentrations and time periods used, BODIPY-cholesterol was not cytotoxic as there was no decrease in the numbers of *B. burgdorferi* during the 6 hr incubation period. Labeled spirochetes did not exhibit any morphological or motility defects in that they maintained their spiral shape and wave-like motion which was demonstrated by several blurry bacteria in the static image (Figure 2C). Furthermore, viability of labeled spirochetes was measured using fluorescent microscopy (Figure 2C). Following incubation of *B. burgdorferi* with 4.0 mg/L BODIPY-cholesterol, spirochetes were reintroduced into BSK-II. The BODIPY-cholesterol did not cause a significant delay in the growth of the spirochetes when compared to controls (Figure 2D).

Given that BODIPY-cholesterol can be incorporated by *B. burgdorferi*, we tested whether the fluorescent cholesterol analog could serve as a substrate for synthesis of cholesterol-glycolipids. The lipids from *B. burgdorferi* incubated with 4.0 mg/L of BODIPY-cholesterol for 4 hrs in BSK-II media lacking free cholesterol were extracted using the Bligh and Dyer solvent extraction method [70] and resolved by chloroform-methanol (85/15) solvent phase on a High Performance Thin Layer Chromatography (HPTLC) plate. Staining of the HPTLC plate with iodine demonstrated that these spirochetes maintain their typical profile (Figure 2E) [11], [12], [30]. UV excitation of the same chloroform-methanol HPTLC plate showed that the BODIPY-cholesterol is incorporated into the cholesterol glycolipids of *B. burgdorferi* (Figure 2E). Free cholesterol and cholesterol-glycolipids (ACGal and CGal) on the plate are visible, but glycolipids that do not contain cholesterol, MGalD and the phospholipids, are not. Furthermore, incubation of BODIPY-cholesterol with *B. burgdorferi* at the beginning of the experiment (no incubation) indicates that the fluorescent cholesterol probe has the same migration profile as cholesterol in our experimental conditions and is not incorporated into the cholesterol-glycolipids as was observed after 4 hrs of incubation with the probe (Figure 2E). We did observe increased levels of CGal labeled with BODIPY-cholesterol when compared to ACGal labeled with the fluorescent probe. It is possible that CGal could serve as a building block or an intermediate species in the synthesis of ACGal and that over the length of these experiments complete synthesis of ACGal is not achieved. Nonetheless, this shows that *B. burgdorferi* used the BODIPY-cholesterol as a substrate for cholesterol-glycolipid synthesis, thus making the individual cholesterol-glycolipids fluorescent when excited by UV light. Because *B. burgdorferi* can incorporate the BODIPY-cholesterol into its cholesterol-glycolipids, we assessed the long term growth and cytotoxicity of the fluorescent cholesterol in BSK-II (Figure 2F). Furthermore, in BSK-II without free cholesterol and without the rabbit serum, the spirochetes with BODIPY-cholesterol do not replicate, but are still alive when viewed using dark field microscopy for up to 96 hrs. This same result is observed when using non-fluorescent cholesterol (Figure 2F). Therefore, BODIPY-cholesterol is similar to non-fluorescent cholesterol in that long-term incubation with the sterols is not cytotoxic to the spirochetes. Furthermore, this experiment indicates that the sterol source does not matter for survival because spirochetes only grow in BSK-II that has to be supplemented with 6% rabbit serum (Figure 2F). We conclude that BODIPY-cholesterol is a useful cholesterol analog in *B. burgdorferi*, and so its behavior is very likely to reflect that of unlabeled cholesterol.

### Exchange of lipids from *B. burgdorferi* to eukaryotic cells

**Figure 3:**
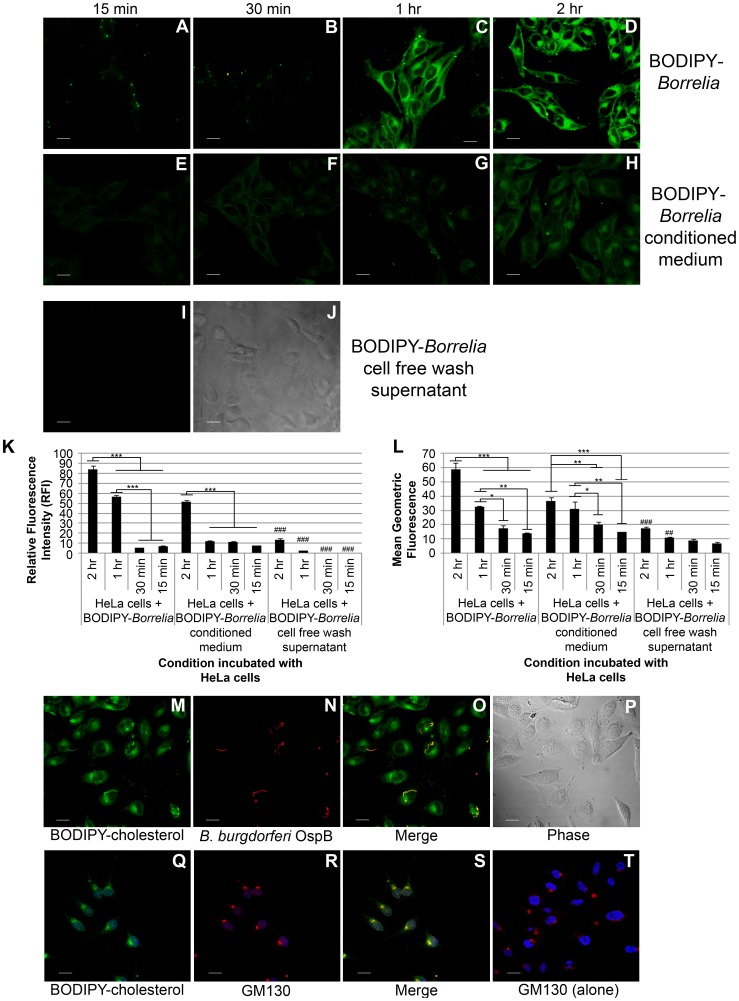
*B. burgdorferi* exchange BODIPY-cholesterol to HeLa cells. *B. burgdorferi* labeled with BODIPY-cholesterol were incubated with HeLa cells for 15 min, 30 min, 1 hr, and 2 hr. **A–D:** HeLa cells exposed to *B. burgdorferi* labeled with BODIPY-cholesterol. **E–H:** HeLa cells exposed to conditioned medium from *B. burgdorferi* labeled with BODIPY-cholesterol. **I–J:** HeLa cells exposed to cell free wash supernatant from *B. burgdorferi* labeled with BODIPY-cholesterol for 2 hrs. **I.** Photograph of negative control representative of all temperatures; **J.** Phase contrast. Scale Bar = 20 µm. **K.** The mean relative fluorescence intensity (RFI) +/− standard error of the mean of HeLa cells from 10 microscope fields were calculated from the different experimental conditions. ANOVA ***p<0.001, ###p<0.001(negative control is significantly less than associated condition). **L.** Mean geometric fluorescence +/− standard error of the mean from three separate flow cytometry analysis of HeLa cells incubated following experimental conditions. ANOVA *p<0.05, *p<0.01 ***p<0.001, ##p<0.01, ###p<0.001(negative control is significantly less than associated experimental condition). **M–P.** Single confocal microscopy optical section illustrates that during the 2 hr incubation the *B. burgdorferi* (yellow) adhered to HeLa cells (green). Scale bar = 20 µm. **Q–T.** *B. burgdorferi* derived cholesterol are processed by the HeLa cell and localize to the Golgi complex. Confocal microscopy demonstrated that the cis-Golgi marker, GM130 (red), co-localizes (yellow) with the BODIPY-cholesterol fluorescence (green). In the control image (**T**) where no *Borrelia* were added, the perinuclear localization (DAPI nuclear stain, blue) of GM130 is consistent with the other images. Scale bar = 20 µm.

The clustering of cholesterol into lipid rafts is a characteristic that is shared by both the eukaryotic host and *B. burgdorferi*. We hypothesized that the presence of cholesterol-rich lipid rafts in both the host and pathogen could serve as an ideal platform for lipid-lipid interactions. There is evidence that *H. pylori* attaches to and partitions in detergent-resistant regions (lipid rafts) of the host cell membrane [62]. To determine whether a direct molecular interaction exists between *B. burgdorferi* and host cells, we performed experiments to observe and quantify if there is any exchange of lipids. HeLa cells were selected for these experiments because *B. burgdorferi* come into contact with epithelial cells during dissemination and infection, and because they are not phagocytic. HeLa cells were grown on glass coverslips and exposed to *B. burgdorferi* labeled with BODIPY-cholesterol at a MOI of 20∶1 at four time intervals at 37°C. In addition to the labeled spirochetes, we incubated the HeLa cells with conditioned medium (after removal of spirochetes labeled with BODIPY-cholesterol) and cell free wash supernatant (as negative control) for the time intervals. Images of HeLa cells incubated with labeled *B. burgdorferi* and the conditioned medium from BODIPY-cholesterol labeled *B. burgdorferi* captured by confocal microscopy show that the lowest levels of transfer occur in the shorter incubation times of 15 min (Figure 3A,E) and 30 min (Figure 3B,F). There appears to be a time dependent threshold between the 30 min and 1 hr incubation times because the labeling is most robust and highest at the longer incubation times of 1 hr (Figure 3C,G) and 2 hrs (Figure 3D,H). In the tested incubation times, we observed little or no transfer in the negative control, the cell free wash supernatant (Figure 3I, J). Time dependency and differences in transfer between labeled spirochetes and conditioned medium were confirmed by RFI analysis (Figure 3K) and flow cytometry (Figure 3L). Furthermore at the 2 hr incubation time the images show that *B. burgdorferi* also come into direct contact with the HeLa cells (Figure 3M–P). This further indicates that the spirochetes make direct contact with the HeLa cells and that this interaction contributes to transfer. These experiments together show that the transfer of *B. burgdorferi* derived lipids occurs through a combination of contact dependent and contact independent mechanisms. Likewise, transfer is time-dependent because the longer the spirochetes interact with the HeLa cells, the more transfer occurs.

To follow the trafficking of BODIPY-cholesterol after incorporation, we used the cis-Golgi marker, GM130 (Figure 3Q–T). Labeled cholesterol trafficked to the Golgi-apparatus within 2 hrs indicating that the cells processed the *B. burgdorferi* derived cholesterol in the same manner as cholesterol from other sources.

To characterize further and understand the process of lipid exchange, we conducted transfer assays using a coincubation time of 2 hrs at different temperatures between 4°C and 37°C. Confocal microscopy demonstrated that at 4°C (Figure S2 A,D) there was significantly less transfer of bacterial derived lipids from labeled spirochetes and conditioned medium when compared to images taken at 25°C (Figure S2 B,E) and 37°C (Figure S2 C,F). The negative control, the cell free wash supernatant, did not show any visible transfer at any temperature (Figure S2 G, H). For RFI measurements (Figure S2 I) and the flow cytometry analysis (Figure S2 J), we observed the same trend when comparing data for two different temperatures where the levels of transferred lipids were significantly higher at the higher temperatures.

**Figure 4:**
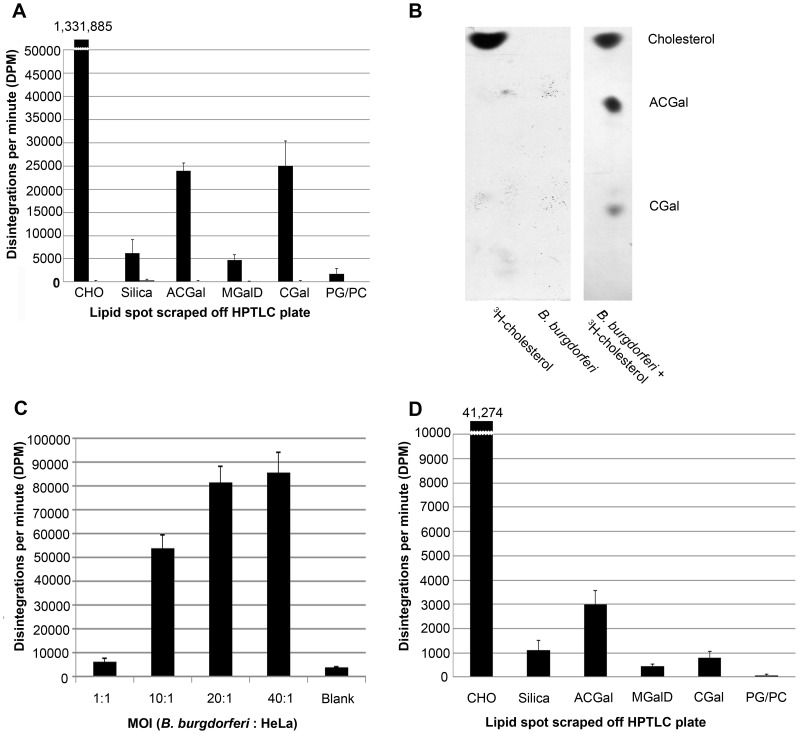
*B. burgdorferi* transfer ^3^H-cholesterol to HeLa cells. **A.** *B. burgdorferi* utilized ^3^H-cholesterol as a substrate for cholesterol glycolipid synthesis; DPM's derived from scraped silica from HPTLC plate. *B. burgdorferi* incubated with ^3^H-cholesterol (black bars) utilized ^3^H-cholesterol as a substrate for cholesterol-glycolipid synthesis and incorporated the radiolabeled sterol into their lipid fraction when compared to unlabeled *B. burgdorferi* (gray bars). **B.** Exposure of the HPTLC plate to Kodak BioMax MR film without scraping demonstrates that the ^3^H-cholesterol was incorporated into the cholesterol-glycolipids. Radioactive cholesterol was not observed in *B. burgdorferi* incubated with unlabeled cholesterol (middle lane). This data supports the results from HPTLC exposed to UV light from Figure 1E. **C.** Spirochetes labeled with 10.0 µCi ^3^H-cholesterol transfer membrane lipids to HeLa cells. Spirochetes were incubated at an MOI of 1∶1, 10∶1, 20∶1, and 40∶1 with HeLa cells for 2 hours at 37°C in BSK-II. HeLa cells were analyzed for transfer by liquid scintillation counting. **D.** *B. burgdorferi* transfer ^3^H-cholesterol labeled glycolipids to HeLa cells. Radiolabeled *B. burgdorferi* were incubated with HeLa cells. The bacteria were removed and the HeLa cell lipid extracts were analyzed by liquid scintillation. Experiments **A, C,** and **D** represent the mean ± standard error of the mean from three separate experiments.

To confirm that the transfer of *B. burgdorferi* lipids to HeLa cells was linked to a specific process and not an artifact created by the addition of the BODIPY fluorophore to the cholesterol molecule, we performed lipid transfer experiments using ^3^H-cholesterol. The increased sensitivity of radiolabeling relative to fluorescence also allowed us to investigate whether cholesterol-glycolipids were transferred to HeLa cells. Lipids from *B. burgdorferi* labeled with ^3^H-cholesterol were extracted using the Bligh and Dyer solvent method [70], and analyzed by HPTLC. The silica from cholesterol lipid spots identified with iodine was scraped from the HPTLC plate and analyzed by liquid scintillation to quantify the amount of ^3^H-cholesterol incorporated into *B. burgdorferi*. As an additional control, silica between the observed bands of free cholesterol and ACGal was also scraped to confirm that each band was distinct. When incubated with ^3^H-cholesterol, *B. burgdorferi* incorporated the radioactive cholesterol into their membrane fraction (Figure 4A). Similar to the incorporation of BODIPY-cholesterol, we found that the spirochetes also utilized the ^3^H-cholesterol as a substrate for synthesis of the cholesterol-glycolipids (Figure 4A). The ratio of ^3^H-cholesterol, ^3^H-cholesterol labeled ACGal, and ^3^H-cholesterol labeled CGal in the spirochete was between 53.5∶1∶1 and 42.6∶1.25∶1 (Figure 4A). We did not observe incorporated radioactivity above background in unlabeled controls or in lipids that do not contain cholesterol, MGalD, phosphatidylcholine, or phosphatidylglycerol (Figure 4A). These data support the fluorescence data already presented in Figure 2E in that the cholesterol is incorporated into the bacterial cholesterol glycolipid fractions because we detected significant incorporation of ^3^H-cholesterol into all cholesterol-glycolipids by HPTLC (Figure 4B).

To demonstrate transfer of lipids from spirochetes to HeLa cells, *B. burgdorferi* labeled with ^3^H-cholesterol were incubated with the cells at a MOI of 40∶1, 20∶1, 10∶1, and 1∶1 for 2 hrs. The HeLa cells were washed three times and lifted from the tissue culture wells. Each condition was measured by liquid scintillation counting to quantify the levels of transfer (Figure 4C). We observed significant disintegrations per minute (DPM) in the higher MOI in a dose-dependent manner (Figure 4C). This agrees with data obtained using BODIPY-cholesterol that there is a transfer of *B. burgdorferi* lipids to the host eukaryotic cells. Additionally, lipids were extracted from HeLa cells following removal of spirochetes and analyzed for their lipid profile by HPTLC (Figure 4D). Evidence that the cholesterol-glycolipids are transferred to the membrane of the HeLa cells comes from identification of the individual cholesterol-glycolipids in the HeLa cell lipid extracts on the HPTLC plate (Figure 4D). Experimental HeLa cell lipid extracts were run on an HPTLC plate in parallel with a reference control *B. burgdorferi* lipid extract. The HPTLC plate was stained with iodine and the bands on the HeLa cell extract samples that corresponded to the known glycolipid bands from the *B. burgdorferi* control lipid extract were scraped for liquid scintillation analysis. The ratio of transferred ^3^H-cholesterol, ^3^H-cholesterol labeled ACGal, and ^3^H-cholesterol labeled CGal was between 56∶4∶1 and 54.67∶5.33∶1 (Figure 4D). It is noteworthy that the ratio of ACGal to free cholesterol in the HeLa cells was higher than that in the spirochetes. This suggests that it transfers more efficiently than cholesterol, and would not be expected if the HeLa cell-associated radioactivity was an artifact of spirochetes attached to the HeLa cells. As an additional control to account for the contribution of spirochetes that remain attached to the cells after washing to the total DPMs, we utilized a GFP-labeled *B. burgdorferi* strain [71] to quantify the percentage of bacteria that did not dissociate from the cells. The HeLa cells were analyzed by the SpectraMaxM2 to calculate GFP fluorescence levels. This control disclosed that 2.2±0.4% (∼61% of the total signal of 3.5±0.9%) could be derived from spirochetes that remained attached. Despite this, however, ∼39% of the signal of ^3^H cholesterol and cholesterol-glycolipid was associated with cells. Furthermore, the observation above that the fraction of radioactive ACGal in HeLa cells after transfer (between 56∶4∶1 and 54.67∶5.33∶1 [cholesterol∶ACGal∶CGal]) was 3 to 5-fold times higher than ACGal derived from radioactively labeled *B. burgdorferi* alone (between 53.5∶1∶1 and 42.6∶1.25∶1 [cholesterol∶ACGal∶CGal]) the amount of ACGal bound to the HeLa cells is 6-fold times higher than that expected if it was just derived from *B. burgdorferi* that had not washed off the HeLa cells. In this study, we have used four different methodologies (fluorescence confocal microscopy, single cell RFI analyses, flow cytometry, and isotope incorporation) to demonstrate lipid exchange from *B. burgdorferi* to cells. Of these four, isotope incorporation was the least robust, but it also provided evidence for lipid exchange through differential incorporation of label and through HPTLC. Thus, using all four experimental approaches, we conclude that there is a transfer or uptake of both cholesterol and the antigenic cholesterol-glycolipids by eukaryotic host cells *in vitro*, and this could be important implications for the pathogenesis of Lyme disease.

### *B. burgdorferi* labeled with BODIPY-cholesterol specifically release fluorescent cholesterol probe in the form of OMV

**Figure 5:**
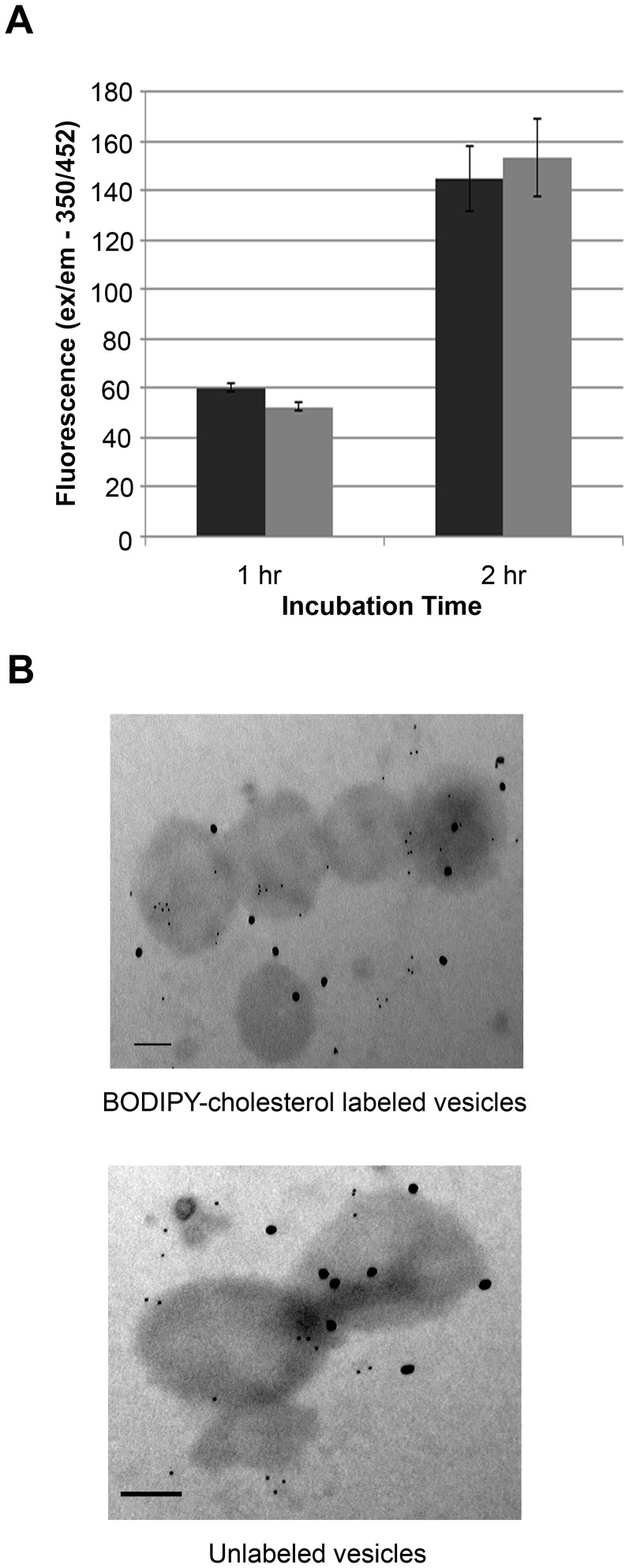
*B. burgdorferi* labeled with BODIPY-cholesterol release the fluorescent cholesterol probe in OMV. **A.** BODIPY-cholesterol labeled and unlabeled *B. burgdorferi* release similar amounts of membrane material. DPH was added to the supernatants collected from *B. burgdorferi* labeled with BODIPY-cholesterol and unlabeled spirochetes to measure released OMV. Using the SpectraMaxM2, the DPH fluorescence was calculated for each sample at the specific time points. Supernatant from labeled *B. burgdorferi* (black); supernatant from unlabeled *B. burgdorferi* (gray). **B.** Transmission electron micrograph showing isolated vesicles from both labeled and unlabeled *B. burgdorferi* OMV. Immunogold labeling of OspB (18 nm) and *B. burgdorferi* glycolipids (6 nm) is seen throughout the vesicles. Scale Bar = 100 nM. Experiment **A** represents the mean ± standard error of the mean from three separate experiments.

Given that HeLa cells acquired BODIPY fluorescence from conditioned medium (Figure 3 E–H), we sought to determine whether free BODIPY-cholesterol was being released nonspecifically or was associated with OMV. To identify released OMV in the supernatants, we used a lipophilic probe, 1,6-diphenyl-1,3,5-hexatriene (DPH). DPH fluoresces in hydrophobic environments such as membranes, but not in aqueous environments and has a linear response with membrane bilayer concentration [72]. By probing the supernatants from BODIPY-cholesterol labeled and unlabeled spirochetes with 1 µg/mL of DPH, we were able to determine if fragments of membrane were being released from the labeled spirochetes and how it compared to release by unlabeled *B. burgdorferi*. Labeled *B. burgdorferi* released intact membrane, in amounts similar to unlabeled *B. burgdorferi* (Figure 5A), suggesting that the membrane release is a natural process and not the result of BODIPY-cholesterol labeling. However, DPH labeling cannot distinguish if membrane release is in the form of an intact OMV. Therefore we analyzed the collected OMV by transmission electron microscopy (TEM). The negative-stain TEM micrographs of isolated vesicles show spherical structures from both labeled and unlabeled *B. burgdorferi* OMV (Figure 5B). In both OMV preparations, we used double immunogold labeling for OspB (18 nm colloidal gold) and the cholesterol-glycolipids (6 nm colloidal gold). Both labeled and unlabeled *B. burgdorferi* release OMV that are similar in morphology and glycolipid content.

**Figure 6:**
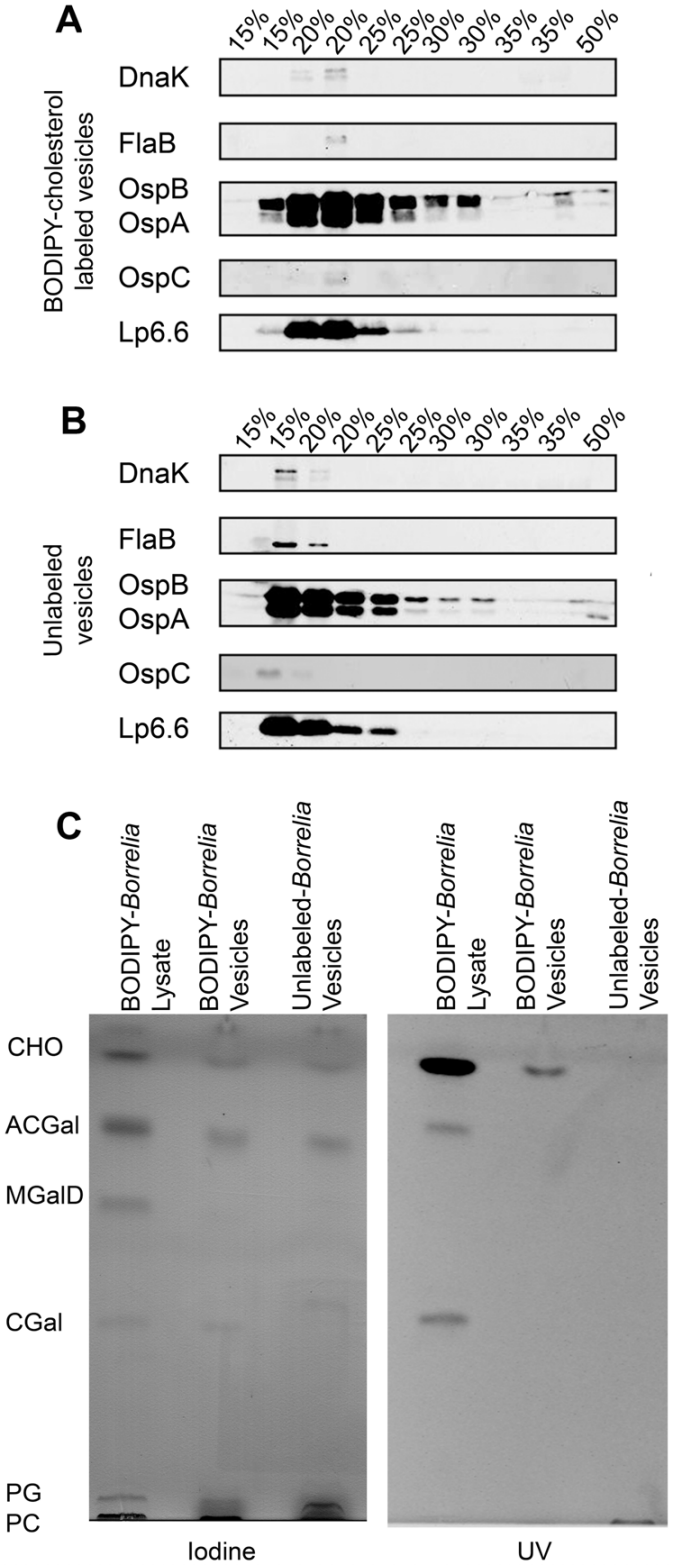
Purified OMV from *B. burgdorferi* contain outer membrane proteins and labeled cholesterol-glycolipids. OMV from labeled and unlabeled *B. burgdorferi* were purified using an Optiprep density gradient. Each percentage (15%–35%) of the discontinuous gradient was collected in two fractions. **A.** SDS-PAGE western blot of the 11 fractions isolated from the supernatants of BODIPY-cholesterol labeled *B. burgdorferi.* Vesicles isolated from fractions 2–5 contain OspB, OspA, and lp6.6. **B.** SDS-PAGE western blot of the 11 fractions isolated from the supernatants of unlabeled *B. burgdorferi*. Vesicles in fractions 2–4 contain OspB, OspA, and lp6.6. **C.** **Left:** Chloroform-methanol (85/15) HPTLC of isolated *B. burgdorferi* OMV. Fractions 15–25% of both labeled and unlabeled OMV were pooled and analyzed for glycolipid content with iodine staining. Labeled and unlabeled OMV have both phospholipids, free cholesterol, ACGal, and CGal. **Right:** UV exposed chloroform-methanol (85/15) HPTLC of isolated *B. burgdorferi* OMV. Vesicles contain the cholesterol-glycolipids and phospholipids, but no MGalD. Vesicles derived from labeled *B. burgdorferi* have the fluorescent cholesterol analog BODIPY-cholesterol.

The release of OMV from the spirochetes (Figure 5B) led us to test whether the OMV play a role in the transfer of lipids to HeLa cells. To confirm that the membranes released from labeled *B. burgdorferi* are OMV and similar in protein content to the unlabeled controls, we purified OMV from labeled and unlabeled *B. burgdorferi* using an Optiprep density gradient. To compare protein content, we performed SDS-PAGE using 11 purified OMV fractions from the supernatants of labeled and unlabeled *B. burgdorferi* and observed similar protein content in the fractions where OMV partition (20–25%). Both preparations had similar distributions of protein across the 11 gradient fractions. Even with the addition of BODIPY-cholesterol to the spirochetes, the OMV are representative of naturally forming vesicles. We further determined by SDS-PAGE and western blot that OspA, OspB, and lp6.6, some of the most abundant lipoproteins in the OMV, are found in both labeled and unlabeled *B. burgdorferi* (Figure 6A and 6B). These electrophoretic and immunoblot data were also supported by previous mass spectrometry data from our laboratory that showed OM proteins were the most abundant in the OMV [73].

### *B. burgdorferi* release OMV that are partially responsible for the transfer of bacterial derived lipids to HeLa cells

In addition to analyzing the protein content of labeled and unlabeled OMV, we also determined their lipid content. To examine the lipids found in *B. burgdorferi* OMV, we pooled fractions 15–25% of both the labeled and unlabeled vesicles. We determined BODIPY-cholesterol labeled and unlabeled OMV have both phospholipids and cholesterol, ACGal, and CGal. The cholesterol-glycolipids are significant lipid components of the vesicles (Figure 6C). To identify fluorescent lipids, the same HPTLC plate was exposed to UV light which demonstrated that the vesicles derived from BODIPY-cholesterol labeled *B. burgdorferi* contain the fluorescent cholesterol analog BODIPY-cholesterol, although in this preparation we were not able to demonstrate BODIPY-cholesterol incorporation into ACGal and CGal (Figure 6C). Detection of fluorescently labeled ACGal and CGal was most likely not observed in the isolated vesicles due to the small amount of material collected and analyzed and the low sensitivity of UV light excitation of the BODIPY-cholesterol probe on the TLC plate. However, the cholesterol-glycolipids were visualized as components of the OMV at the single molecule level using the 6 nm colloidal gold anti-rabbit secondary antibody and TEM (Figure 5B).

**Figure 7:**
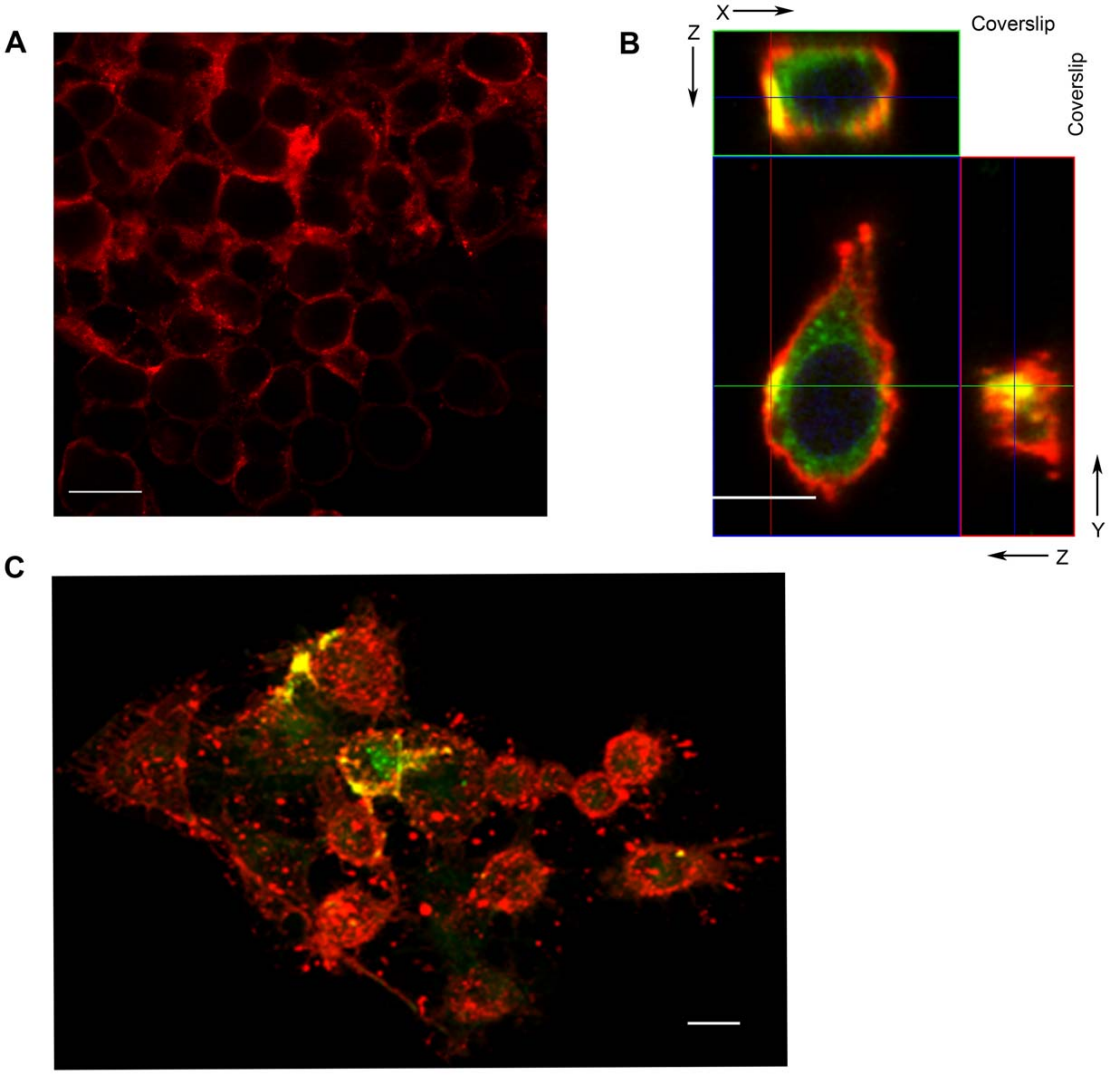
OMV from *B. burgdorferi* labeled with BODIPY-cholesterol contribute to the transfer of bacterial lipids to HeLa cells. Vesicles purified from *B. burgdorferi* labeled with BODIPY-cholesterol were incubated with adherent HeLa cells for 2 hrs. **A.** Vybrant Cell Labeling Solution DiI labels the plasma membrane of HeLa cells. **B.** Single image of OMV labeled with BODIPY-cholesterol and Vybrant Cell Label Solution DiI. Incubation of labeled OMV (green) colocalize (yellow) at the plasma membrane (red). **C.** Merged three dimensional confocal micrograph demonstrate Vybrant Cell Labeling Solution DiI (red) is located at the plasma membrane of the HeLa cell. There is colocalization (yellow) of labeled OMV (green) on the surface of the HeLa cells. Scale Bar = 20 µm.

To test for the possibility that the OMV released by *B. burgdorferi* could be responsible for transfer of bacterial antigens to the HeLa cells, we isolated vesicles from *B. burgdorferi* labeled with BODIPY-cholesterol. The HeLa cells were washed with PBS and incubated with Vybrant Cell-Labeling Solution DiI to label their plasma membranes (Figure 7A). We observed that when isolated OMV labeled with BODIPY-cholesterol are incubated with HeLa cells in the transfer assay, there is a colocalization between the BODIPY-cholesterol probe and the Vybrant Cell Labeling Solution DiI probe on the surface of the HeLa cells. We observed this colocalization at the single cell level (merged cross section, Figure 7B) and in merged 3D composite image of multiple cells on the cover slip (Figure 7C). Similar to the transfer assays conducted with the whole bacteria, we observed internalization in the form of free BODIPY-cholesterol fluorescence inside of the HeLa cell. This was represented by diffuse staining inside the plasma membrane of the composite images. These confocal micrographs show that there is an exchange of bacterial derived lipids from *B. burgdorferi* to eukaryotic cells and that this transfer can be executed by OMV.

## Discussion

Through the use of fluorescent and radiolabeled cholesterol, we showed that a lipid exchange between spirochetes and host cells can occur. Two main conclusions can be derived from our studies. First, we show that when *B. burgdorferi* come in direct contact with epithelial cells the spirochetes can extract cholesterol from epithelial cell membranes. Second, both cholesterol and the antigenic cholesterol-glycolipids of *B. burgdorferi* are transferred to epithelial cells through direct contact between the spirochete and the plasma membrane and through released OMV.

As an extracellular pathogen, nutrient acquisition from the immediate environment is an essential process for *B. burgdorferi* to be able to persist in the host. Here we provide evidence that *B. burgdorferi* can extract cholesterol, an essential membrane lipid, from eukaryotic cells. We demonstrated that *B. burgdorferi* attach to epithelial cells and can incorporate cholesterol directly from the plasma membrane. Using fluorescence microscopy, *B. burgdorferi* were shown to attach to epithelial cells and associate with BODIPY-cholesterol labeled regions of the plasma membrane. Evidence that the spirochetes extract cholesterol from epithelial cells comes from confocal microscopy images which showed colocalization of the BODIPY-cholesterol from the HeLa cells and the lipoprotein OspB at the point of attachment, and also the presence of BODIPY-cholesterol throughout the spirochete which extended away from the cell. Cholesterol acquisition has been shown to also be an important process for extracellular pathogens. Cholesterol and cholesterol-glycolipids comprise a significant portion of the bacterial membrane of *H. pylori*. Using similar techniques to ours, *H. pylori* was shown to attach to cholesterol-rich domains and acquire cholesterol from eukaryotic membranes [61], [62]. For some obligate intracellular pathogens, such as *Coxiella burnetii* and the *Chlamydia* species, lipid acquisition is also essential to establish and maintain an active infection [74], [75] and an exogenous source of lipids is necessary for their growth [6], [74], [75]. In intracellular bacteria, phospholipids and cholesterol along with machinery to synthesize these lipids are trafficked to the inclusion vacuole containing these bacteria [76]–[79]. Understanding how bacteria use or alter these lipids is an area of active research [80]. Together, these findings represent diverse mechanisms for cholesterol acquisition.

The host antibody response to *B. burgdorferi* has been studied extensively, but the understanding of the role of the unique cholesterol-glycolipids in pathogenesis is limited. Antibody responses to these glycolipids have been detected throughout the course of Lyme disease [9], [11], [12], [20]. To observe the role of cholesterol and cholesterol-glycolipids of *B. burgdorferi*, we developed methods to incorporate labeled cholesterol into the spirochetal membranes and showed that the labeled cholesterol is used as a substrate for synthesis of the cholesterol-glycolipids. This allowed us to track the cholesterol-glycolipids through several experimental approaches.

In addition to the ability of *B. burgdorferi* to tolerate and use labeled cholesterol as a substrate for cholesterol-glycolipid synthesis, we also demonstrated that the cholesterol and cholesterol-glycolipids are transferred to the epithelial cells. After incubation with epithelial cells, evidence from confocal microscopy showed that fluorescently labeled cholesterol and cholesterol-glycolipids were observed in the plasma membrane as well as Golgi apparatus. The presence of the fluorescent cholesterol in the Golgi apparatus indicated that the eukaryotic cells are processing and trafficking all or part of the exogenous BODIPY-cholesterol in a manner similar to that used for cholesterol derived from other sources [64]. To bolster the results from the fluorescence experiments, we also used radioactive cholesterol to demonstrate lipid exchange. With the higher sensitivity of radiolabeled cholesterol, we were able to establish that not just labeled cholesterol, but also labeled ACGal and CGal, the two antigenic cholesterol glycolipids, can be transferred to epithelial cells and are present in the eukaryotic membrane. Because of the increased sensitivity of the experiments using radiolabeled spirochetes, we were able to detect low but significant DPM values in the epithelial lipid extracts, suggesting that there is an exchange of the antigenic cholesterol-glycolipids into the HeLa cells. Because the values obtained from the transfer experiments are generally low, we used several different approaches to demonstrate lipid exchange, including spectrophotometry, flow cytometry, quantifiable fluorescence microscopy (RFI), and liquid scintillation. Further documentation of transfer was obtained at the level of the single cell using fluorescent and confocal microscopy. Regardless of the amount of transfer, together, our results demonstrated that a spirochetal cholesterol based antigen is transferred to and present in the membrane of the epithelial cells.

We showed that length of coincubation time and the temperature of the coincubation between the spirochetes and the epithelial cells can change the amount of overall transferred lipid. In addition, we investigated how OMV can contribute to the transfer of lipids between the spirochetes and host cells. *Borrelia* OMV have been isolated [81]–[83] and extensively studied [84]–[89]. Our method to collect and isolate OMV from *Borrelia* sought to collect OMV in BSK-II from live organisms, thus replicating the same experimental conditions would be found in as the conditioned medium. We demonstrated that transfer can occur through released OMV from the spirochete, and the vesicles can attach to or be incorporated into the epithelial plasma membrane. Furthermore, we determined that the cholesterol-glycolipids were significant components of the OMV, and similar to other species of *Borrelia* [81], the isolated OMV were also rich in OM proteins.

We have shown that *B. burgdorferi* extract cholesterol from the plasma membrane of eukaryotic cells and that cholesterol-glycolipids can be transferred to epithelial cell membranes by a contact dependent mechanism through direct attachment. These two events, cholesterol acquisition and transfer of antigenic lipids, might not be mutually exclusive. One possible explanation for the contact dependent transfer could be that the spirochetes are required to attach to the eukaryotic plasma membrane to acquire cholesterol. Uncharacterized spirochetal transferases [18] potentially associated with the OM could also extract cholesterol from the host cells for synthesis of the cholesterol-glycolipids. During this event, it is possible that the cholesterol-glycolipids are left behind in the plasma membrane of the epithelial cell. There may also be the mechanism in which cells acquire these spirochetal lipids via released OMV that are rich in cholesterol-glycolipids [30], [73]. The ability of OMV from pathogenic bacteria to participate in host-pathogen interactions as virulence factors has been well documented [90]. Furthermore, there is evidence that OMV from other bacteria can fuse with the cell membrane [91]–[93]. We demonstrated that OMV derived from fluorescently labeled *B. burgdorferi* can transfer the fluorescent cholesterol to the epithelial cells. Therefore, the OMV of *B. burgdorferi* could serve as a vehicle to transfer the cholesterol-glycolipids, fuse with the cell membrane, and act as virulence factors that influence and modulate the host immune response.

In summary, using fluorescent and radiolabeled cholesterol, we have documented that *B. burgdorferi* extract cholesterol from the plasma membrane of eukaryotic cells and that prokaryotic cholesterol-glycolipids can be transferred to epithelial cell membranes by two mechanisms (i) a contact dependent mechanism through direct attachment and (ii) a contact independent method through released OMV. The *B. burgdorferi* membrane is unique in that it contains lipid rafts, with cholesterol and cholesterol-glycolipids with physical properties that are similar to those of eukaryotic membranes. Transfer of antigenic cholesterol-glycolipids could play a major role in the pathogenesis of the spirochetoses. Given the limited biosynthetic capabilities of *B. burgdorferi* to make cholesterol and other important lipids, the process of cholesterol extraction from host cells is likely to be more biologically significant for the nutrition of the spirochetes early in infection as nutrient acquisition is crucial for the replication of the bacteria. Once the bacteria have disseminated and an infection has been established, it is likely that even at low levels, the transfer of antigenic lipids from the spirochete to host cells becomes more significant. Whether inserted directly into the plasma membrane of eukaryotic cells, or attached to the surface of these cells, the presence of foreign antigens with similar composition and structural characteristics could have multiple consequences for the host immune response. It is also possible that these transferred lipids could contribute to heightened inflammation and arthritis. Furthermore, if the immune response were to recognize cells with transferred lipid antigens, the cells themselves become targets of immune effectors.

## Materials and Methods

### Bacteria, cultures, and loading of fluorescent and radioactive cholesterol analogs

*B. burgdorferi* strain B31 were grown in microareophilic conditions in BSK-II medium [94] supplemented with 6% rabbit serum (Sigma) at 33°C. For the incorporation experiments, a BSK-II without free cholesterol was made by using cholesterol free CMRL-1066 (Invitrogen). Removal of cholesterol from the CMRL-1066 eliminated the free cholesterol found in BSK-II (0.2 µg/mL. When the BSK-II is supplemented with 6% rabbit serum, the final concentration of cholesterol in the cholesterol-free BSK-II was 0.78 µg/mL. The environment sensitive fluorescent cholesterol analog, 23-(dipyrrometheneboron difluoride)-24-norcholesterol (TopFlour Cholesterol or BODIPY-cholesterol, Avanti Polar Lipids), was added to BSK-II without free cholesterol medium for 6 hrs at a concentration of 0.2 mg/L, 2.0 mg/L, and 4.0 mg/L of BODIPY-cholesterol. After the 6 hr incubations, the bacteria were washed three times with Hank's balanced salt solution (HBSS, Gibco). Fluorescence readings were calculated using a SpectraMaxM2 (ex/em - 490/504). The fluorescently labeled *B. burgdorferi* were used immediately for all assays.

*B. burgdorferi* were also labeled with ^3^H-cholesterol (American Radiolabeled Chemicals, Inc.). Spirochetes were washed three times with HBSS and the endogenous cholesterol was depleted using 10 mM MβCD. After depletion, the cholesterol was replaced by incubating the spirochetes with 4.0 mg/L of cholesterol and 10.0 µCi of ^3^H-cholesterol in HBSS. *B. burgdorferi* were washed three times with HBSS and were immediately used for all assays.

### Lipid extraction, purification, and analysis

*B. burgdorferi* were washed three times in large volumes of HBSS, before lipid extraction using the Bligh and Dyer method [70]. The lipid extracts were concentrated under constant nitrogen gas stream. Lipid extracts were separated by thin-layer chromatography on Si250 HPTLC silica plates (J.T. Baker) with chloroform/methanol (85/15) and visualized with iodine vapor staining or exposure to UV light. Lipid extracts from samples containing ^3^H-cholesterol were separated by HPTLC and stained with iodine vapor. The spots on the plate were scraped and the silica containing the radioactive cholesterol was analyzed by liquid scintillation counting. For direct visualization of the HPTLC plate, the plate was first sprayed with EN^3^HANCE Spray (DuPont) and exposed using BioMax MR Film (Kodak) for 4 and 14 days at −80°C and developed using a Medical Film Processor Model SRX-101A (Konica).

### Optimization for fluorescent labeling of *B. burgdorferi* membrane

*B. burgdorferi* labeled with BODIPY-cholesterol were analyzed by the SpectraMax M2 plate reader (ex/em - 490/504) for incorporation of fluorescence. The viability, morphology, and motility of the BODIPY-cholesterol treated *B. burgdorferi* were assessed by dark-field enumeration and fluorescence microscopy. The lipids of *B. burgdorferi* labeled with BODIPY-cholesterol were isolated and resolved on an HPTLC Si250 silica plate with chloroform-methanol (85/15) and exposed with UV light and stained with iodine vapor for visualization of the lipids. Standards were known R_f_ values from identical solvent systems [11], [12], [30]. *B. burgdorferi* incubated with BODIPY-cholesterol were also analyzed for their ability to grow or recover following incorporation of the fluorescent label. Labeled *B. burgdorferi* were washed in HBSS, and resuspended in BSK-II medium, and growth was assessed by dark-field enumeration.

### *B. burgdorferi* lipid transfer assay

HeLa cells were maintained in DMEM medium (Gibco) with 10% fetal calf serum (Pel-Freez). The HeLa cells were grown on glass coverslips in T175 tissue culture flasks (Falcon). Cells were infected with *B. burgdorferi* labeled with BODIPY-cholesterol at a multiplicity of infection (MOI) of 20∶1 for 2 hrs. Conditioned medium and negative control cell free wash supernatant were also added to the coverslips for 2 hrs. Conditioned medium supernatants were generated by incubating *B. burgdorferi* labeled with BODIPY-cholesterol in BSK-II for 2 hrs. The spirochetes were pelleted by centrifugation at high speed for 15 min and the supernatants (cell-free) were examined by dark field and fluorescent microscopy to ensure that intact organisms were not present. The supernatants were added directly to the HeLa cells for 2 hrs. The cell free wash supernatant was included as a negative control to ensure that the BODIPY-cholesterol is not loosely associated with the OM or released nonspecifically from *B. burgdorferi* as there was no observable transfer of label. The negative control or cell free wash supernatant was generated by resuspension of the labeled *B. burgdorferi* pellet after the final wash in BSK-II, to the HeLa cells for 2 hrs. The coverslips were washed three times in phosphate buffered saline (PBS, Gibco), fixed in 2.5% paraformaldehyde and blocked with 1% bovine serum albumin (Sigma) in PBS for immunofluorescence staining. The remaining *B. burgdorferi* that were attached to the HeLa cells were detected with CB2, a murine monoclonal antibody to OspB [95] followed by an Alexa Fluor 594 goat anti-mouse IgG (Invitrogen).

The samples analyzed for BODIPY-cholesterol colocalization with the cis-Golgi complex were probed with a monoclonal rabbit GM130 antibody (Abcam) followed by a Texas Red goat anti-rabbit IgG (Abcam). Samples were imaged by confocal laser microscopy using a Zeiss LSM 510 META NLO Two-Photon Laser Scanning Confocal Microscope System.

Additional approaches were used to detect lipid exchange between *B. burgdorferi* and cells. RFI of the HeLa cells from 10 microscope fields of vision were calculated using Zeiss LSM 510 META NLO Two-Photon Laser Scanning Confocal Microscope System Software. The mean geometric fluorescence was calculated from samples that were analyzed by a FACScan/Calibur for BODIPY fluorescence.

For experiments using radiolabeled *B. burgdorferi*, HeLa cells were incubated with *B. burgdorferi* labeled with 10.0 µCi ^3^H-cholesterol ^3^H-cholesterol at a MOI of 40∶1 for 2 hrs. The HeLa cells were extensively washed to remove the spirochetes. HeLa cells were lifted from the tissue culture flasks using 0.05%Trypsin/EDTA (Gibco). Transferred radioactivity to the HeLa cells was detected by extracting the lipids using the Bligh and Dyer solvent extraction method. Lipids were separated on a HPTLC plate using chloroform-methanol (85/15). The HPTLC plate was stained with iodine and spots on the plate were scraped. In addition, incorporation of isotope into the lipids was measured by scraping the silica from the HPTLC plate, analyzed by liquid scintillation using a Beckman LS 6500 Liquid Scintillation Counter and reported as DPM.

To control for *B. burgdorferi* that remained attached to HeLa cells after washing, *B. burgdorferi* that constitutively expressed GFP [71] was utilized. The GFP expressing *B. burgdorferi* were incubated with HeLa cells at a MOI of 40∶1 for 2 hrs. The HeLa cells were extensively washed to remove the spirochetes. HeLa cells were lifted from the tissue culture flasks using 0.05%Trypsin/EDTA (Gibco). Using the SpectraMaxM2, the amount of GFP fluorescence (*B. burgdorferi* still attached to HeLa cells) was measured in the HeLa cell pellet.

### Quantification of released BODIPY-cholesterol from *B. burgdorferi*

For detection of released membrane material, labeled *B* and unlabeled *B. burgdorferi* were incubated in HBSS for 2 hrs. The supernatants were collected at 1 hr and 2 hrs. The supernatants were labeled with 1 µg/ml of the hydrophobic-sensitive, fluorescent, lipophilic probe DPH (Invitrogen) for 30 min at 33°C. After 20 min, the supernatants were analyzed for fluorescence in a SpectraMax M2 plate reader using an excitation of 360 nm and emission of 430 nm.

### Vesicle isolation and purification

*B. burgdorferi* labeled with BODIPY-cholesterol or unlabeled *B. burgdorferi* in the late-log phase of growth were pelleted by centrifugation, and resuspended in fresh BSK-II media. To keep similar incubation times as the transfer assay, the labeled and unlabeled spirochetes were incubated for 2 hrs at 37°C to collect released vesicles. Following the removal of spirochetes, the supernatants were filtered twice using 0.22 µm-pore-size Steriflip filters (Millipore). Crude membrane and outer membrane vesicles (OMV) in the filtered supernatant were concentrated by ultracentrifugation for 1 hr at 100,000× g. To purify the OMV, the membrane pellet was resuspended in 60% OptiPrep (Axis Shield). A discontinuous gradient was made following the manufacturer's instructions. The discontinuous gradient was centrifuged for 16 hrs at 100,000× g. The OMV were concentrated to form a white band that floated to the interface between the 20% layer and the 25% layer of the gradient. The gradient fractions were collected in 1 mL volumes (two 1 mL fractions for each OptiPrep gradient percentage) from the top of the tube with the least dense faction being collected first. The OMV from fraction 15%, 20%, and 25% were pooled based on similar protein contents to maximize the amount of OMV collected [73]. The isolated OMV were concentrated and removed from the OptiPrep solution by centrifugation for 1 hr at 100,000× g. The pelleted, purified OMV were resuspended in 20 mM HEPES (pH 7.5). The OMV were immediately used for vesicle transfer assays.

### *B. burgdorferi* outer membrane vesicle transfer assay

HeLa cells were grown on glass coverslips in 24 well tissue culture plates. Cells were incubated with 40 µg (based on protein content) of vesicles purified from BODIPY-cholesterol labeled *B. burgdorferi* for 2 hrs. Protein content was determined by BCA Assay and a Coomassie Plus Assay (Pierce). The HeLa cells were washed with HBSS to remove the vesicles. To label the plasma membrane of the HeLa cells, the coverslips were then incubated with Vybrant Cell Labeling Solution DiI (Invitrogen) following the manufacturer's instructions. The coverslips were fixed with 2.5% paraformaldehyde and were imaged by confocal laser microscopy using a Zeiss LSM 510 META NLO Two-Photon Laser Scanning Confocal Microscope System.

### *B. burgdorferi* cholesterol extraction from epithelial membranes

HeLa cells were grown on glass coverslips in 24 well tissue culture plates. The HeLa cells were preloaded with 5 µg/mL of BODIPY-cholesterol for 2 hrs. To remove excess BODIPY-cholesterol, the cells were washed twice with 5 mg/mL of methyl-β-cyclodextrin (MβCD, Sigma) and one final time with BSK-II. HeLa cells labeled with BODIPY-cholesterol were incubated in BSK-II for 1 hr, before the supernatants and cells were measured for BODIPY-cholesterol fluorescence using the SpectraMax M2.

To observe the cholesterol extraction from HeLa cell membranes, the cells were infected at an MOI of 40∶1 for 1 hr washed 3 times with PBS and fixed with 2.5% paraformaldehyde for 15 min. *B. burgdorferi* were detected by incubation with CB2 hybridoma supernatants [95] followed by an Alexa Fluor 594 goat anti-mouse IgG (Invitrogen). The cells were imaged by confocal laser microscopy using a Zeiss LSM 510 META NLO Two-Photon Laser Scanning Confocal Microscope System.

### Statistics

Statistics were calculated using GraphPad InStat 3 (GraphPad Software).

## Supporting Information

Figure S1 ***B. burgdorferi*** **attach to HeLa cells and acquire cholesterol from the epithelial cell membranes.** HeLa cells incubated with BODIPY-cholesterol (green) and washed with MβCD were incubated with *B. burgdorferi* (red) for 1 hr at an MOI of 40∶1. Cells were fixed, stained with CB2 (red) and examined by confocal fluorescence microscopy. Colocalization (yellow) on the single confocal micrographs indicates the *B. burgdorferi* acquired the fluorescent cholesterol. Scale bars = 10 µm.
(TIF)

Figure S2 **Transfer of lipids from** ***B. burgdorferi*** **to HeLa cells is specific and temperature dependent.** *B. burgdorferi* labeled with BODIPY-cholesterol were incubated with HeLa cells at 37°C, 25°C, and 4°C for 2 hr. **A–C:** HeLa cells exposed to *B. burgdorferi* labeled with BODIPY-cholesterol for 2 hrs. **A.** 4°C; **B.** 25°C; **C.** 37°C, same image as Figure 3D. **D–F:** HeLa cells exposed to conditioned medium from *B. burgdorferi* labeled with BODIPY-cholesterol for 2 hrs. **D.** 4°C; **E.** 25°C; **F.** 37°C, same image as Figure 3H. **G–H:** HeLa cells exposed to cell free wash supernatant from *B. burgdorferi* labeled with BODIPY-cholesterol for 2 hrs. **G.** Photograph of negative control representative of all temperatures; **H.** Phase contrast. Scale Bar = 20 µm. **I.** The mean relative fluorescence intensity (RFI) +/− standard error of the mean of HeLa cells from 10 microscope fields were calculated from the different experimental conditions. ANOVA ***p<0.001, ###p<0.001(negative control is significantly less than associated condition). **J.** Mean geometric fluorescence +/− standard error of the mean from three separate flow cytometry analysis of HeLa cells incubated following experimental conditions. ANOVA *p<0.05, **p<0.01, ***p<0.001, ###p<0.001 (negative control is significantly less than associated experimental condition).
(TIF)
