# Supplementary figures and images for: Lipid Exchange between Borrelia burgdorferi and Host Cells
Source: PLoS Pathog. 2013 Jan 10;9(1):e1003109. doi: 10.1371/journal.ppat.1003109 (PMC3542181; doi:10.1371/journal.ppat.1003109)

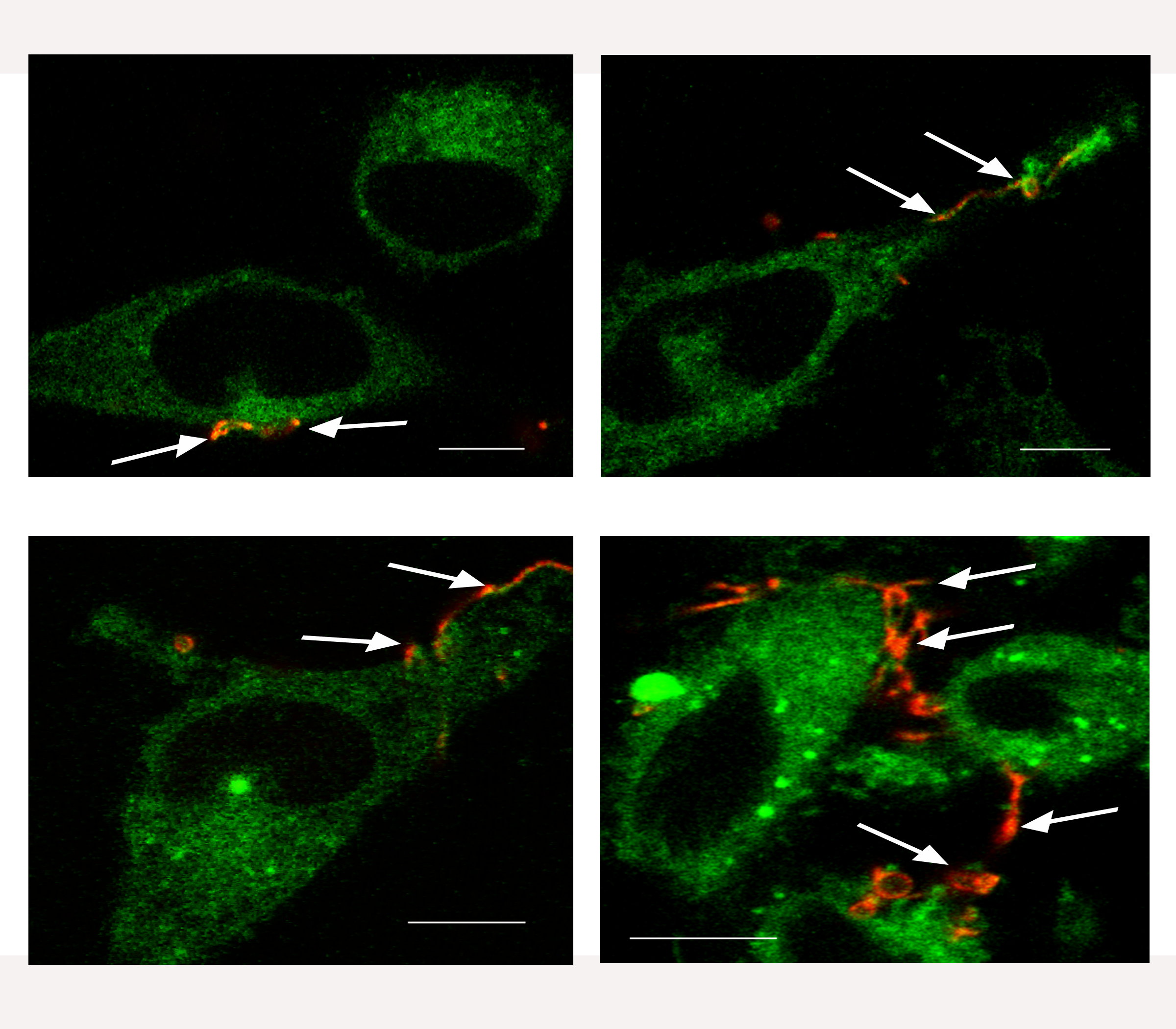

Supplement: Figure S1 — B. burgdorferi attach to HeLa cells and acquire cholesterol from the epithelial cell membranes. HeLa cells incubated with BODIPY-cholesterol (green) and washed with MβCD were incubated with B. burgdorferi (red) for 1 hr at an MOI of 40∶1. Cells were fixed, stained with CB2 (red) and examined by confocal fluorescence microscopy. Colocalization (yellow) on the single confocal micrographs indicates the B. burgdorferi acquired the fluorescent cholesterol. Scale bars = 10 µm. (TIF) [file ppat.1003109.s001.tif]

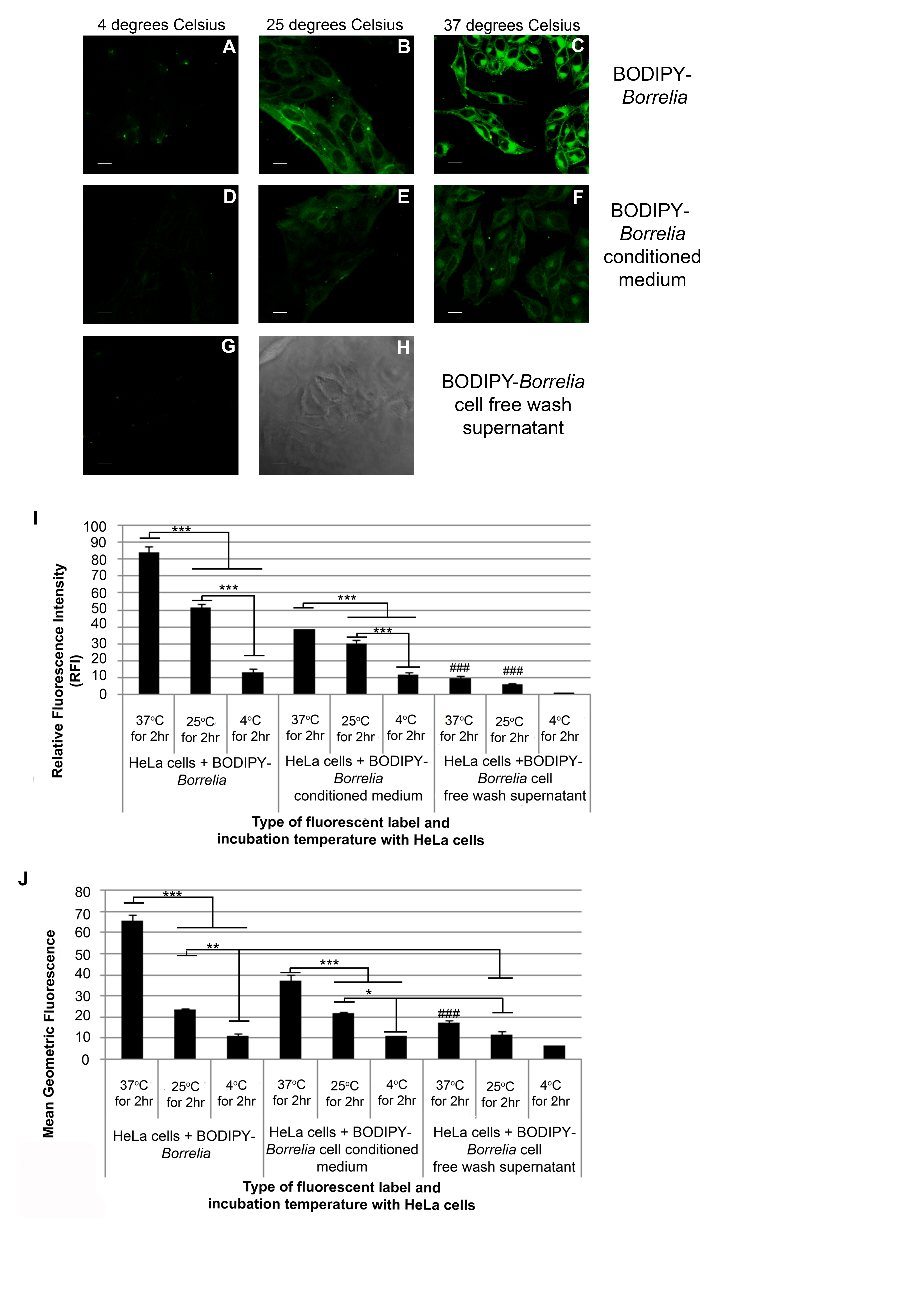

Supplement: Figure S2 — Transfer of lipids from B. burgdorferi to HeLa cells is specific and temperature dependent. B. burgdorferi labeled with BODIPY-cholesterol were incubated with HeLa cells at 37°C, 25°C, and 4°C for 2 hr. A–C: HeLa cells exposed to B. burgdorferi labeled with BODIPY-cholesterol for 2 hrs. A. 4°C; B. 25°C; C. 37°C, same image as Figure 3D. D–F: HeLa cells exposed to conditioned medium from B. burgdorferi labeled with BODIPY-cholesterol for 2 hrs. D. 4°C; E. 25°C; F. 37°C, same image as Figure 3H. G–H: HeLa cells exposed to cell free wash supernatant from B. burgdorferi labeled with BODIPY-cholesterol for 2 hrs. G. Photograph of negative control representative of all temperatures; H. Phase contrast. Scale Bar = 20 µm. I. The mean relative fluorescence intensity (RFI) +/− standard error of the mean of HeLa cells from 10 microscope fields were calculated from the different experimental conditions. ANOVA ***p<0.001, ###p<0.001(negative control is significantly less than associated condition). J. Mean geometric fluorescence +/− standard error of the mean from three separate flow cytometry analysis of HeLa cells incubated following experimental conditions. ANOVA *p<0.05, **p<0.01, ***p<0.001, ###p<0.001 (negative control is significantly less than associated experimental condition). (TIF) [file ppat.1003109.s002.tif]
